# Tandem domain swapping: determinants of multidomain protein misfolding

**DOI:** 10.1016/j.sbi.2019.05.012

**Published:** 2019-10

**Authors:** Aleix Lafita, Pengfei Tian, Robert B Best, Alex Bateman

**Affiliations:** 1 European Molecular Biology Laboratory, European Bioinformatics Institute, Wellcome Genome Campus, Hinxton, Cambridge, UK; 2 Novozymes A/S, Krogshøjvej 36, DK-2880 Bagsværd, Denmark; 3 Laboratory of Chemical Physics, National Institute of Diabetes and Digestive and Kidney Diseases, National Institutes of Health, Bethesda, MD, USA

## Abstract

Tandem homologous domains in proteins are susceptible to misfolding through the formation of domain swaps, non-native conformations involving the exchange of equivalent structural elements between adjacent domains. Cutting-edge biophysical experiments have recently allowed the observation of tandem domain swapping events at the single molecule level. In addition, computer simulations have shed light into the molecular mechanisms of domain swap formation and serve as the basis for methods to systematically predict them. At present, the number of studies on tandem domain swaps is still small and limited to a few domain folds, but they offer important insights into the folding and evolution of multidomain proteins with applications in the field of protein design.

## Highlights

• Domain swapping refers to the exchange of structural elements between protein domains.
• Experiments show that tandem homologous domains are prone to domain swapping.
• Recent studies establish a framework to understand the formation of tandem domain swaps.
• Prediction of tandem domain swaps is possible but hindered by the amount of available data.

## Graphical abstract

**Figure:**
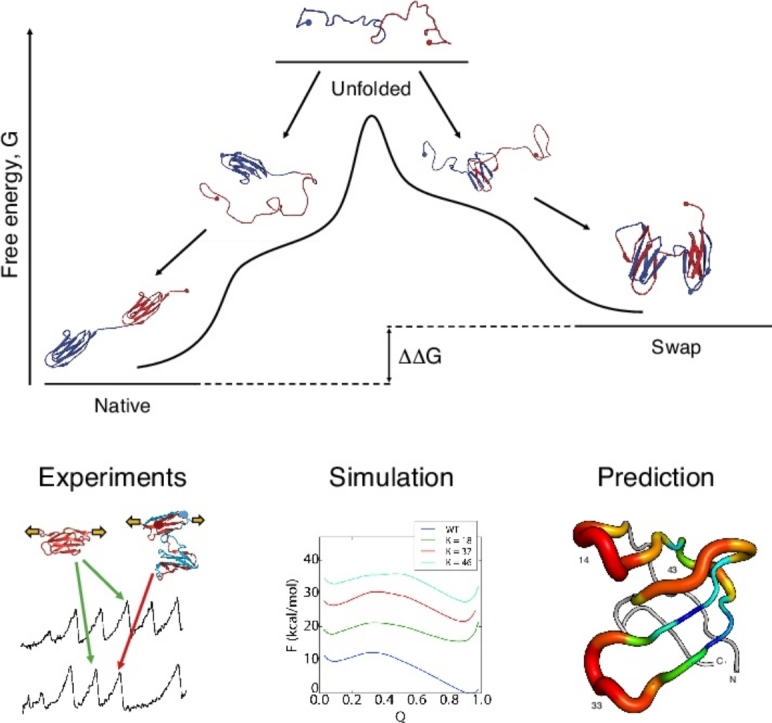

**Current Opinion in Structural Biology** 2019, **58**:97–104

This review comes from a themed issue on **Biophysical and computational methods**

Edited by **Alan R Lowe** and **Laura S Itzhaki**

For a complete overview see the Issue and the Editorial

Available online 28th June 2019

**https://doi.org/10.1016/j.sbi.2019.05.012**

0959-440X/© 2019 The Authors. Published by Elsevier Ltd. This is an open access article under the CC BY license (http://creativecommons.org/licenses/by/4.0/).

## Introduction

The formation of distinct globular structural units, called domains, during the folding of protein chains has been an active research topic since the early days of structural biology [1]. Although protein domains are generally observed to fold independently, misfolding interactions can occur between adjacent domains in multidomain proteins [2]. These types of interactions have been shown experimentally to be more prevalent if the tandem domains are homologous, owing to the similarity of their sequences [3^•^,^4^]. Two plausible mechanisms by which similar sequences might interact are via the formation of parallel, in-register β-sheet structures (amyloid-like), or by the reciprocal exchange of equivalent secondary structure elements, forming a metastable domain-swapped conformation [5^••^,6^••^]. Amyloid-like protein misfolding is well studied and has been extensively reviewed, most recently in Dobson *et al.* [7]. Here, we focus on the formation of tandem domain swap misfolds. Despite the limited amount of experimental data on tandem domain swapping, computer simulations have established a framework to understand the molecular mechanisms of their formation and systematically predict their prevalence in multidomain proteins [5^••^,8^••^,9^••^]. In this review, we present the state of the art experimental techniques and computational methods to study and predict tandem domain swap misfolding and discuss their challenges and applications.

## Misfolding in tandem repeat domains

**Figure 1:**
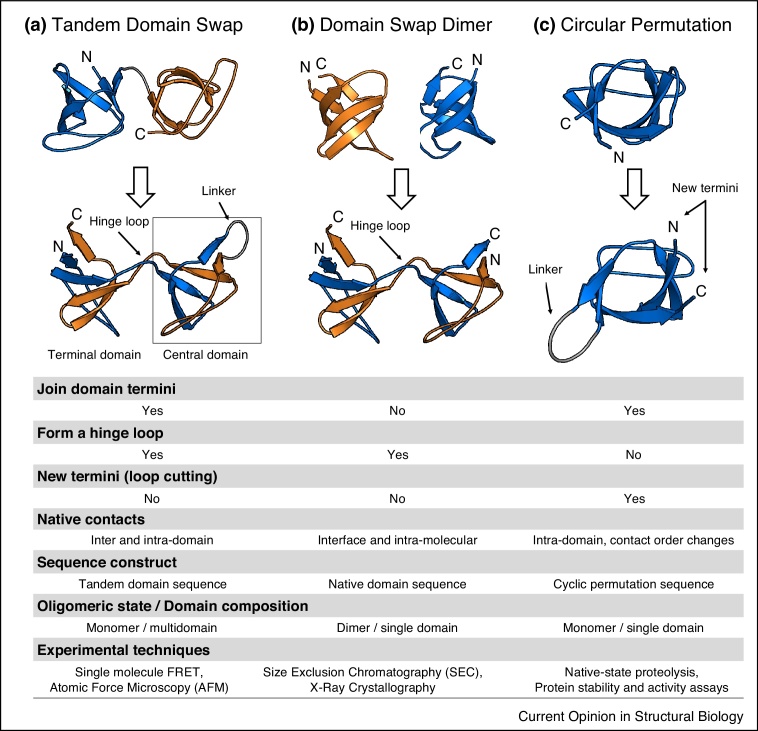
Comparison of structural conformation and features of **(a)** tandem domain swaps, **(b)** domain-swapped dimers and **(c)** circular permutations. The ‘central domain’ of a tandem domain swap is equivalent to a circular permutation, formed from the central region of the two-domain sequence comprising the C-terminus of the first domain and the N-terminus of the second domain, joined by the inter-domain linker. The ‘terminal domain’ comprises the remaining parts of the sequence and folds with the termini in their native location. The ‘hinge loop’ that connects the central and terminal domains is characteristic of domain swapping. Other features and common experimental techniques that have been used thus far to study each of the domain folding variants are listed in the lower table. Structures shown are models of an SH3 domain from simulations by Tian and Best [8^••^].

Folding of a protein chain into its functional three-dimensional structure requires the formation of so-called native interactions, that is, energetically favourable contacts between specific residues of the chain. When two identical domains are spatially close, these native contacts can potentially be satisfied within domains (intra-domain) or across domains (inter-domain) [10^•^]. Therefore, non-native conformations where domains are folded one onto the other in an intertwined topology can potentially also satisfy the native domain interactions [5^••^], as depicted in Figure 1a,b. Domain swaps have been extensively studied as part of protein oligomerization, a topic that has been reviewed on multiple occasions [11,12^•^,^40^], but had not been considered in the context of multidomain misfolding until recently.

The structure of each domain within a domain-swapped oligomer is essentially identical to the native fold and is characterised by the same backbone dihedral angles (Figure 1b). The only important differences are in the region which opens up to link the two domains, known as the ‘hinge loop’. The properties of the hinge loop, such as length and sequence composition, have been established as critical for the propensity to domain swap. The most notorious is perhaps that prolines in hinge loops favour domain swapping [13]. Attempts to engineer hinge loops in protein domains have been largely successful [14,15]. Although there does not seem to be a unified mechanism for domain swapping [16], in some cases complete unfolding has been shown to be a requirement [17]. A wide variety of proteins have now been shown to oligomerise as domain swaps, including all-α, all-β and mixed α/β folds [11], with several hundred experimental structures deposited in the Protein Data Bank (PDB) [18].

Closer analysis of tandem domain swaps also reveals fundamental similarities to so-called circular permutations (Figure 1c), cyclic rearrangements of a domain sequence that fold into the same structural unit [19]. Circular permutations occur frequently in natural proteins [20]. Protein structures seem remarkably insensitive to circular permutations, since most viable (i.e. folded) variants retain stability and activity [21^•^]. However, it is important to note that only a fraction of the possible circular permutations of any protein domain will be folded, as will become clear. Circular permutations have been useful to resolve the folding mechanisms of protein domains in a number of studies [23, 24, 25,42]. This has resulted in thousands of experimental structures of circularly permuted variants of different domain folds available in the PDB [41].

We are now in a position to better describe tandem domain swaps, which can be broken down into features of circular permutations and domain swaps. It is clear from the illustration in Figure 1a that both structural domains in the misfolded state comprise regions of both the N-terminal and C-terminal domains in the native sequence — hence they are obviously domain-swapped. Furthermore, of the two newly formed domains, one (that formed from the central region of the sequence) is effectively a circular permutant (the ‘central domain’). The other ‘terminal domain’ is domain-swapped, but conserves the termini of the native fold. Unlike circular permutations and domain-swapped oligomers, there are at present no experimental structures of tandem domain swaps deposited in the PDB. However, recent experiments have provided strong evidence for their formation.

## Experimental evidence for domain-swapped misfolding

**Figure 2:**
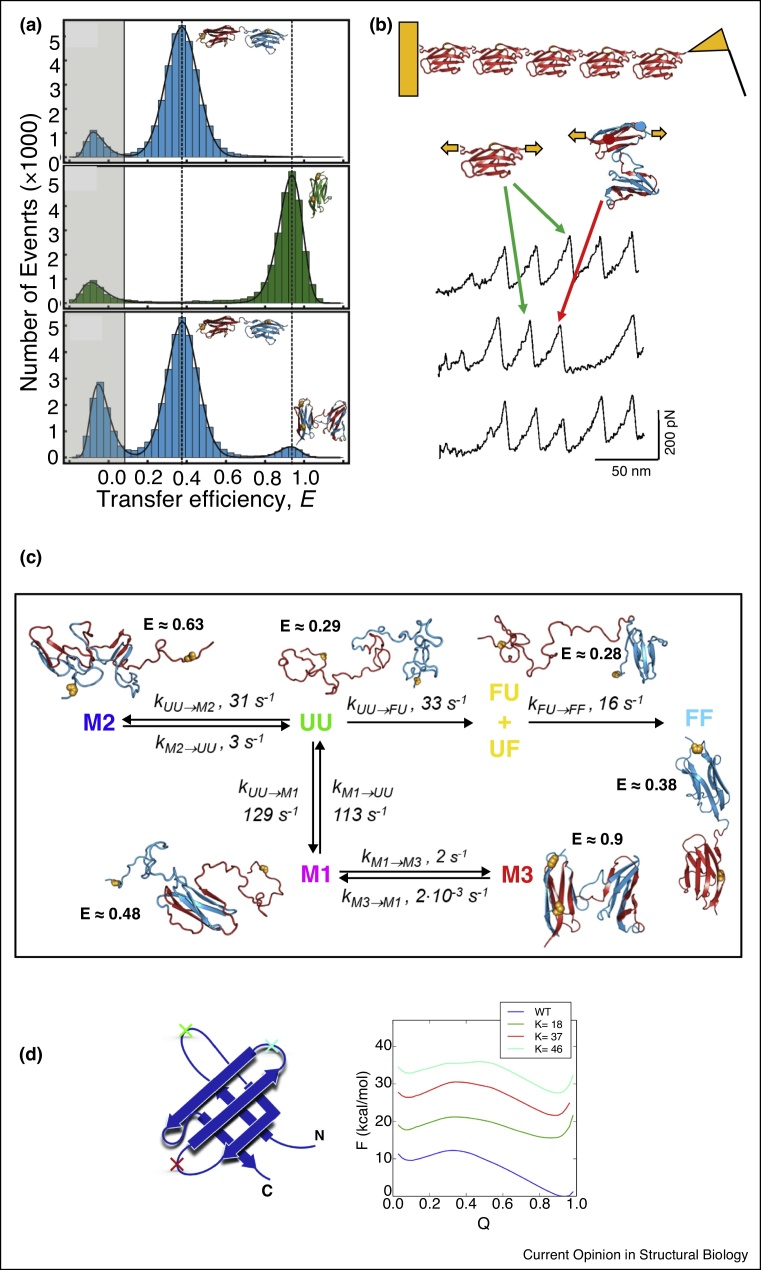
Experimental evidence for misfolding in tandem domain repeats. **(a)** Single molecule FRET of a tandem dimer of titin I27 with one label in each of the N-terminal and C-terminal native domains yields low FRET efficiency (top). After unfolding into chemical denaturant and refolding, a second peak appears with a high FRET efficiency (bottom) that matches that of a doubly labelled single titin domain (center). **(b)** AFM experiments pull a construct of titin I27 domains from the ends. This normally yields a regular array of force peaks, each corresponding to the unfolding of a single I27 domain (green arrows). Occasionally a misfolding event with spacing double that of a regular unfolding event is observed (red arrow). **(c)** Time-resolved FRET experiments have revealed a complex mechanism for the formation of domain-swapped dimers. An unfolded chain can proceed via three pathways — formation of a native tandem repeat (species FF), formation of a domain-swapped dimer (species M3), or formation of an amyloid-like misfold (species M2). The amyloid like species is however much more transient than the others, with a lifetime of seconds versus weeks or longer for M3 or FF. **(d)** Molecular dynamics simulations have been used to evaluate the most stable domain swap variants, something that cannot be resolved using single-molecule FRET or AFM experiments. In the case of the SH3, the domain swap with the hinge loop at sequence position *K* = 18 is the most stable of the three possibilities evaluated in Tian and Best [8^••^]. Reaction coordinate Q represents the fraction of native contacts. Subfigures reproduced with permission from (a) Borgia *et al.* [27^••^], (b) Oberhauser *et al.* [26^•^], (c) Borgia *et al.* [6^••^] and (d) Tian and Best [8^••^].

The first direct evidence of tandem domain misfolding came from Atomic Force Microscopy (AFM) experiments on tandem repeats of identical immunoglobulin-like I27 domains of titin [26^•^]. In this work, stretching the protein after allowing it to refold sometimes revealed the existence of structures exhibiting the same resistance to force as the native single domain structure, but which led to an increase in molecular length corresponding to two domains (Figure 2b). This finding suggested a stable misfolded state (possibly native-like) involving interactions between two domains. The phenomenon was not restricted to identical adjacent domains, as this same study showed a similar misfolding occurring between domains of a native fragment of the extracellular matrix protein tenascin [26^•^]. However, the nature of the misfolded state was not clear at the time.

More recently, single molecule Förster Resonance Energy Transfer (FRET) studies have been used to examine misfolding in tandem repeats of I27 [6^••^,27^••^], in which the FRET labels were attached near the termini of the protein. Upon refolding of a tandem dimer of I27 from a chemically denatured state, both the native state with a low FRET efficiency and a second state with a high FRET efficiency were observed (Figure 2a). The FRET study also provided specific structural evidence for the nature of the domain-swapped misfolded state. The FRET transfer efficiency, which reflects the distance between the FRET labels, was the same when the labels were attached to residues *i* and *j* in a single titin domain as in the misfolded tandem dimer when the labels were conjugated instead to residues *i* and *j* in each of the adjacent domains of a titin dimer [27^••^]. This finding by itself was very suggestive of a structure involving domain swapping, as was the longevity of the misfolded state.

Further time-resolved FRET experiments later revealed a complex mechanism for the formation of the domain-swapped misfolds [6^••^]. In addition to partially folded species on pathway to the native and domain-swapped state, an additional misfolded state, attributed to amyloid-like species was also identified (Figure 2c). However, this was only stable for a short time, likely due to the greater stability of native-like and domain-swapped structures, and the limited number of repeats available.

So far, titin I27 domains have been the prototype for studies of tandem domain misfolding, but other recent experiments with these techniques suggest it is applicable to other domain folds, for example, in crystallins [28]. Although these experiments can detect the presence of domain-swapped species, they are not able to resolve which domain-swapped conformation is the most probable. Computer simulations have been used for this purpose.

## Molecular simulations on tandem domain swapping

Molecular dynamics simulations have long been used to explore the folding mechanisms of proteins, including the viability of circular permutations [29] and the formation of domain-swapped oligomers [10^•^,^30^]. In the case of multidomain protein misfolding, coarse grained simulations were able to rationalise the experimental FRET and AFM observations in terms of a structural model [27^••^]. As is the case for intermolecular domain swapping, the domain-swapped misfolded proteins are also stabilised by contacts which are analogous to those formed in the native fold. This makes it possible to use energy functions based on the native structure, or ‘Gō models’, to model their formation [10^•^,27^••^]. Further simulation studies using a combination of a sequence-based energy function with a structure-based one showed that the presence of tandem identical domains introduces additional frustration into the folding energy landscape, that is, additional minima in the folding free energy landscape that can trap the protein into metastable misfolded states, predicting an ‘amyloid-like’ misfolded state in addition to the domain-swapped misfolded state [5^••^].

Simulations have also proved useful in rationalizing why some tandem domains appear prone to domain-swapped misfolding, while others do not. In a more recent study, Tian and Best [8^••^] compared the stability of different tandem domain-swapped variants across a set of representative domain folds (Figure 2d). Using a structure-based simulation model, they were able to correctly predict cases in which domain-swapped misfolding was known to occur, as well as those where it was not. Unexpectedly, the misfolding did not seem to correlate with the rate of formation of the central domain, or with proxies for the folding rate such as contact order [31]. Rather, they found that the stability of the central domain was a more important criterion, which they were able to rationalise in terms of an energy landscape model [8^••^]. The stability of this domain could be well approximated by a simple alchemical scheme involving two steps, removing the need to run molecular simulations. These steps involve the formation of a loop from the inter-domain linker and the unfolding of a loop in the native domain to form a hinge loop, named join and cut respectively. This observation has served as the basis for the development of a tandem domain swap prediction method [9^••^].

## Systematic prediction of tandem domain swapping

The prediction of tandem domain swaps is fundamentally similar to the prediction of circular permutations and domain-swapped oligomers, which have been explored before. For circular permutations, prediction methods have centered on the identification of viable permutation positions in the domain sequence that would become the new termini [32,22]. Similarly, domain swap oligomer predictions have focused on the feasibility of forming hinge loops in a domain structure [33^•^,^34^]. Graph theoretical approaches on the residue interaction network calculated from native domain structures are the most common, although some methods also use other additional information to improve the performance [22]. The results are presented as sequence profiles, where the propensity to be involved in a circular permutant or domain swap is assigned to each residue along the protein chain. Such representation has proven useful to identify the number and location of stable swaps and permutants. Unfortunately, only the CPred method is available to the public and as a web server [22], hindering large-scale bioinformatics analysis.

**Figure 3:**
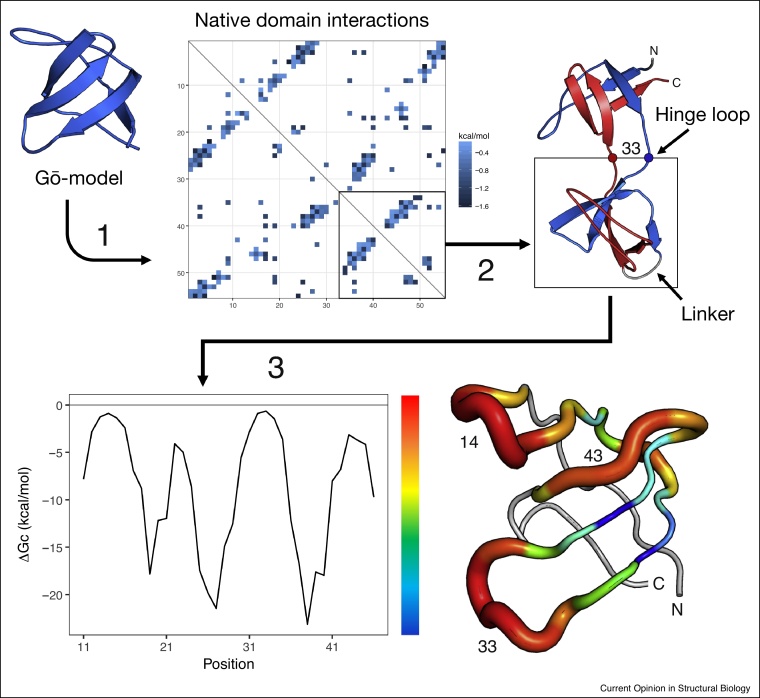
Steps of the alchemical free energy estimation procedure in TADOSS, used to predict the tandem domain swap propensity from the structure of a single protein domain: (1) the energy of the native residue contacts in the domain is calculated using a Gō model, (2) the energy contributions of forming a hinge loop and connecting the domain termini with a linker are estimated from the distorted native contacts, and (3) a free energy profile along the domain sequence is constructed by systematically evaluating all possible hinge loop positions, where higher free energy differences correspond to more stable hinge loops. The energy profile can be mapped in 3D to the structure of the domain to visually identify hinge loop hotspots.

Building from the insights in the simulations by Tian and Best [8^••^], we recently introduced a new computational tool named TADOSS that estimates the relative free energy of native and domain-swapped variants by means of an ‘alchemical’ path between the structures [9^••^]. That is, rather than considering the physical (but intractable) pathway of unfolding one structure and refolding it onto another, the domain-swapped structure is created by an artificial pathway of cutting and joining the chain. The alchemical free energy for this transformation, $\text{ΔΔ}G_{alchemical}$, is based on a Gō-like model for the folding of a single domain, and is divided into the free energy changes incurred by cutting ($\text{Δ}G_{C}$) and joining ($\text{Δ}G_{J}$) the chain to form the circular permutant central domain of the domain-swapped misfolded state (Figure 3). The inherent assumption is that the stability of the terminal domain is similar to that of a native fold, so the central domain explains most of the difference in stability.

The direct estimation of the free energy allows the comparison of stabilities across different domains and is therefore more generally applicable than the estimation of relative probabilities. Furthermore, the distinction between the different steps (join and cut) allows the rationalization of predictions in terms of molecular determinants. For example, we can see in Table 1 that the linker connecting the domain termini is one of the most significant contributors to stability in GB1 and ubiquitin domains. Hence, inter-domain linkers are predicted to have a large effect on the misfolding propensity and should be targeted by protein engineering.

**Table 1:** Comparison of alchemical free energy predictions by TADOSS for a few representative domains used by Tian and Best [8^••^]. Contributions from each term of the energy function are separated in columns 3, 4 and 5 using different combinations of parameters L (linker length) and p (hinge loop length) and combined in columns 6, 7 and 8 to estimate the prevalence of each domain folding variant: circular permutants (CP) involve columns 3 and 4 (join and cut), domain swap (DS) dimers involve column 5 only (hinge) and tandem DS involve columns 3 and 5 (join and hinge). All values are free energies in kcal/mol.

Domain	PDB code	Δ*G* join termini (*L* = 0)	Δ*G* cut loop (*p* = 0)	Δ*G* hinge loop (*p* = 3)	ΔΔ*G* CP	ΔΔ*G* DS dimer	ΔΔ*G* tandem DS
GB1	1PGA	−16.7	3.8	−1.9	−12.9	−1.9	−18.6
UBQ	1UBQ	−13.4	3.8	−2.4	−9.6	−2.4	−15.8
I27	1TIT	−9.7	5.2	−0.5	−4.5	−0.5	−10.2
FN3	1TEN	−7.6	3.8	−2.0	−3.8	−2.0	−9.6
PDZ	2VWR	−5.6	7.8	2.2	2.2	2.2	−3.4
SH3	1SHG	−3.0	5.3	−0.3	2.3	−0.3	−3.3
SH2	1TZE	−1.8	6.7	1.0	4.9	1.0	−0.8

Thanks to the modularity of the alchemical free energy, the method is also applicable to evaluate circular permutations and domain-swapped oligomers with minor changes in the parameters (Table 1). For example, the alchemical free energy correlates well with experimental ΔG measurements of DHFR circular permutations characterised by Iwakura *et al.* [23], hinge loop positions from experimentally determined domain swap dimers used for method evaluation by Ding *et al.* [33^•^], and predictions by CPred. The TADOSS method is open source and has been made available for free to use by anyone under an open MIT license.

## Future perspectives

Determinants of misfolding in multidomain proteins are not yet fully understood. The studies presented here cover one type of misfolding event involving the formation of domain swaps in tandem homologous domains. Efforts from the experimental and computational side have established principles to understand the molecular mechanisms involved in tandem domain swapping, but the amount and diversity of available data are still limited. Nevertheless, the similarity of tandem domain swaps to other well studied domain folding variants, circular permutations and domain swapping in protein oligomerization, has in part permitted complex molecular simulations and their systematic prediction.

One of the most evident gaps is the lack of experimentally solved structures of a tandem domain swap. There are sensible reasons for this absence, such as the low prevalence of tandem identical domain repeats in natural proteins, the selective reduction of repeats in crystallization constructs, or the transient nature and heterogeneity of tandem domain swap conformations. However, such a structure would be very helpful to set a precedent for future studies on tandem domain swaps. Given that the misfolded tandem domain-swapped state is in equilibrium with the native folded state, it will be necessary to design protein sequences that stabilise the misfolded conformation. One possibility might be the use of disulfide bridges, although this is not without complications. Alternatively, it should be possible to selectively introduce mutations which stabilise a specific swapped state in detriment of the native state. A possible avenue for rational design that has been successful in the past may be the use of models from coevolutionary sequence information [35]. Other experimental techniques could also be used to capture folding intermediate states with high resolution, for instance pressure jump NMR [36] or multicolour single-molecule FRET [37].

On the prediction side, the TADOSS method presented here only accounts for protein topology, that is, it predicts *folds* which are likely to be prone to undergo tandem domain-swapping. Furthermore, TADOSS works under the assumption that the tandem domains are of identical sequence, but we know that the very large majority of tandem homologous domains are non-identical. For native multidomain proteins, in which the adjacent domains are homologous but not identical, it is known that the sequences of adjacent domains influence misfolding propensity [3^•^,27^••^]. Thus, inclusion of sequence effects will be an important direction for future prediction algorithms, such as simple contact potentials from evolutionary sequence analysis. Future developments could make use of available high-throughput experimental data on the stability of circular permutants. Other features are also important for the formation of tandem domain swaps but have not yet been explored as predictors, like the additional contacts formed in hinge loops or the rigidity and preferred conformations of loops and linkers.

Protein misfolding via tandem domain swapping can present major problems for the design and production of novel protein constructs. For instance, to increase avidity of interactions of designed protein biotherapeutics it would be convenient to simply duplicate the active part of protein. However, this may lead to increased protein misfolding if the sequence is not optimised to avoid tandem domain misfolds. Domain swapping has also been associated with the formation of amyloid fibrils involved in protein deposition diseases in the context of protein oligomerization [38], from which similar consequences for tandem domain swapping could follow. In conclusion, tandem domain swapping is an active research area with many open challenges. Improvements in our understanding of this phenomenon will not only improve our knowledge of protein folding and misfolding diseases, but also improve our ability to produce better protein therapeutics.

## Funding

This work was supported by the intramural research program of the National Institute of Diabetes and Digestive and Kidney Diseases (grant number ZIA DK075104-06) of the National Institutes of Health to P.T. and R.B.B. This work was funded by the European Molecular Biology Laboratory.

## Conflict of interest statement

Nothing declared.

## References and recommended reading

Papers of particular interest, published within the period of review, have been highlighted as:

• of special interest
•• of outstanding interest
